# Transient Phenomena in Gene Expression after Induction of Transcription

**DOI:** 10.1371/journal.pone.0035044

**Published:** 2012-04-23

**Authors:** Carlus Deneke, Sophia Rudorf, Angelo Valleriani

**Affiliations:** Department of Theory and Bio-Systems, Max Planck Institute of Colloids and Interfaces, Potsdam, Germany; University of Sheffield, United Kingdom

## Abstract

When transcription of a gene is induced by a stimulus, the number of its mRNA molecules changes with time. Here we discuss how this time evolution depends on the shape of the mRNA lifetime distribution. Analysis of the statistical properties of this change reveals transient effects on polysomes, ribosomal profiles, and rate of protein synthesis. Our studies reveal that transient phenomena in gene expression strongly depend on the specific form of the mRNA lifetime distribution.

## Introduction

Together with DNA replication and transcription, translation of mRNA is one of the fundamental processes in cells. Indeed, the fidelity of translation and the speed of ribosomes ensure correct and reliable protein delivery. Yet the process of mRNA degradation governs the reaction time of the cell to changing environmental conditions. One can obtain a deeper understanding of the dynamics of protein synthesis only by considering the time scales that govern the dynamics of polysomes [1]–[3], the sequence (or codon) dependent elongation speed of the ribosomes [4]–[6], and the effect of mRNA stability on polysomes [2] and on the synthesis of proteins [7].

This manuscript is a contribution to our understanding of transient phenomena in gene expression. Here we describe theoretically the time dependent balance between transcription and mRNA degradation. We consider a population of cells under balanced growth conditions, such as those considered theoretically in [8] and often pursued in experiments: Under these conditions the total number of cells is in balance between growth and dilution, the cell size distribution is stationary, all external growth conditions are also constant in time, and the cells are not synchronized.

In many experiments, the transcription of genes placed on recombinant plasmids within the cells is induced by specific drugs. Therefore, conclusions about translation and protein expression depend on the time of measurement after the induction. A similar effect is observed also in certain natural systems. One example is given by the reaction of the adaptive immune system T-cells to an appropriate stimulus [9].

It is known that mRNAs are degraded by different biochemical pathways both in prokaryotes and in eukaryotes [10]. In addition, measurements of the decay of the mRNA amount [11]–[13] have shown that many decay patterns do not follow an exponential behavior [12], [13]. Indeed, the clustering of decay patterns in ref. [13] reveals that at most 117 out of 1102 mRNA species decay more or less exponential. On the one hand, the non-exponential behavior of the other mRNAs could in principle be due to the perturbing nature of the experimental technique. On the other hand, we believe that the non-exponential behavior is rather a consequence of the complexity in the biochemistry of mRNA degradation [10]. Yet, in absence of more precise experiments, one cannot discern the contribution of these two possibilities.

We will first assume that transcription of one chosen gene is induced at time zero with a constant transcription rate 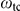 per cell. In those cases in which transcription is not identical between cells or even if transcription is varying stochastically, we will assume that 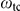 is the average transcription rate in a large sample of cells. Furthermore, we consider the fact that the lifetime 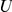 of each mRNA is random and that it is distributed according to the probability density 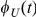. The multitude and complexity of degradation mechanisms lead to a large variety of mRNA lifetime distributions. The theory developed in this article holds for any form of the lifetime probability density 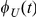. However, in the following, we will consider two different exemplary cases of 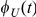, namely on the one hand the exponential lifetime density
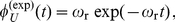
(1)with average value 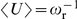. A straightforward extension of Eq. (1) is the gamma density
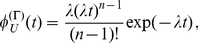
(2)with average value 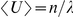. Note that for 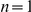 the gamma density reconstitutes the exponential density.

Whereas the lifetime density 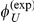 describes the decay of mRNA species in a simple first-order kinetic model, the density 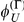 is related to a more refined model of mRNA decay where multiple successive biochemical steps are required for degradation. Moreover, the decay related to a gamma lifetime distribution of the mRNAs with general shape parameter 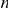 can in principle describe the patterns found in the majority of the clusters found in ref. [13].

**Figure 1:**
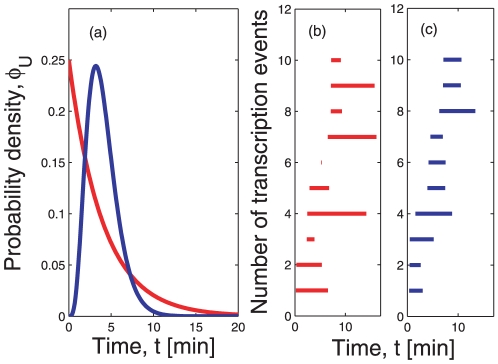
Lifetime densities and growth of the number of mRNAs. Panel (a) shows the exponential lifetime density 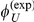 (red) defined in (1) and the gamma density 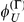 with 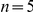 (blue) defined in (2) for equal average lifetimes 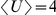 min. The latter distribution is more narrow since the variance scales with the inverse of the shape parameter 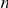. This difference in the variance plays a role in panels (b) and (c) where we show examples of a simulation of the process of creation and degradation of mRNA. Each horizontal bar represents one mRNA and the mRNAs originate at random time points according to a Poisson process. The length of each bar represents the lifetime of the mRNA and for each bar the length is drawn at random according to the distributions 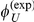 (panel (b)) and 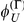 (panel (c)). Clearly, the variation of the length of the bars stems from the different variance of the distributions shown in panel (a). One can follow the growth of the number of mRNAs with time by counting, at each point in time, how many mRNAs are present at that point in time. This is given by the number of horizontal bars that are crossed by a vertical line at time 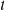. In this and in all following plots the origination of new mRNA molecules occurs at a constant rate 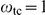 mRNA per minute per cell for simplicity.

In the following, to be able to better compare the two degradation modes we set 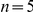 in (2) and fix 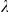 and 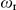 such that the average lifetimes 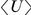 are identical for both distributions. The two exemplary lifetime densities are depicted in Fig. 1a. Note that the particular choice of 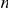 is not decisive for the results. To see this, in Fig. S1 we compare the effect of different shape parameters 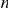 on the lifetime density and the time evolution of the number of mRNAs. Moreover, in order to emphasize certain transient effects, we will compare short-living mRNAs with mRNAs that are more stable by choosing different average lifetimes 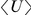. To our knowledge, all previous works on modeling of gene expression have assumed an exponential lifetime distribution with constant decay rate such as in Eq. (1) [14]–[16]. If one instead considers the density (2), or any other non-exponential density, one departs from the simple mathematical framework based on the memoryless (or Markov) property connected to the exponential distribution and one needs to implement advanced techniques in stochastic processes that we have treated in detail in section *Models and Methods*. To illustrate our results we have chosen the relevant parameters such that they match the experimental values of *E. coli*. Nevertheless, our conclusions hold for eukaryotic cells as well. Experimentally, the challenge consists in the simultaneous determination of the synthesis and decay kinetics. A promising method is based on metabolic labeling [17], however a high temporal resolution is required to accurately determine the lifetime distribution and transients related to mRNA expression.

## Results and Discussion

Starting from zero amount of mRNA of a given gene, after the induction of transcription there is an increase of the number of mRNA molecules. This process eventually leads to a stationary state, which reflects the balance between synthesis and degradation of mRNA. However, even if the average transcription rate per cell 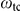 is constant, the patterns of growth of the number of mRNAs depend on the choice of the lifetime density 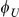. To illustrate this process, Fig. 1, panels (b) and (c), shows the first few minutes of two randomly chosen realizations of the process of mRNA growth. Clearly, the growth according to the exponential lifetime density 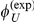 depicted in Fig. 1b produces mRNAs with a broader lifetime span than the gamma distribution 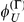 shown in Fig. 1c. This becomes evident if one considers the variance of the two distributions. Whereas the variance of the exponential distribution is 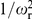, for the gamma density it becomes 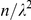. Thus, for equal average lifetimes 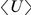, the variance of a gamma density is smaller by a factor of 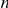 as compared to the exponential case. For the exemplary case with shape parameter 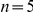 this can be seen also in Fig. 1a.

### Time dependent distribution of mRNAs

**Figure 2:**
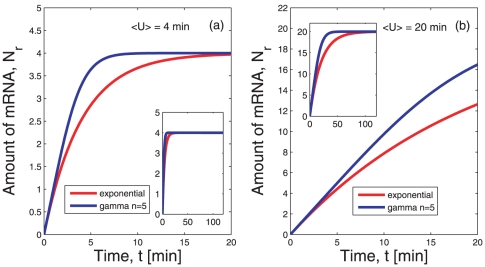
Growth of the average number of mRNAs over time. The red lines represent 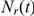 from Eq. (6) when the lifetime density is exponential and is given by Eq. (1). The blue lines depict 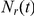 when the lifetime density is gamma-like, as in Eq. (2). Panel (a) shows the growth of the number of mRNAs under the two different lifetime densities when the average lifetime is 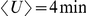, which is the estimated average lifetime in *E. coli* cells [11]. Here the stationary state is reached earlier with the gamma distribution 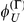 than with the exponential distribution 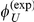. In both cases, the stationary state is reached before twenty minutes. Panel (b) shows the behavior of 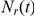 for the exponential and the gamma distributions if the average lifetime is 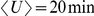, which would correspond to long-living mRNAs in *E. coli*. Even twenty minutes after induction, the average level of expression per cell depends on the form of the lifetime density. The inset of panel (b) shows that the steady state mRNA level is reached after about two hours.

The distribution of the number of mRNAs at time 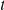 after the start of transcription is given by
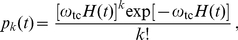
(3)where
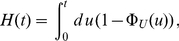
(4)and 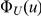 is the probability distribution of 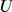 as given by
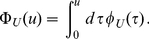
(5)The density 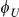 can be any probability density, in particular one of the densities 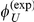 or 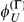. Note that (3) is a time dependent Poisson distribution with parameter 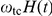. Thus, the average number 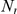 of mRNA molecules can be easily written as
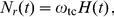
(6)which can be followed in time in Fig. 2.

The time scale to reach a steady state depends critically on two aspects. On the one hand, the average lifetime of the mRNA 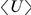 plays an important role, as for larger 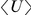 the steady state is reached at a later point. On the other hand, the time to steady state is strongly influenced by the specific form of the lifetime density 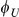 (Fig. 1 and Fig. S1). This fact has several implications that will be investigated in the next sections.

As 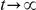, the number of mRNAs reaches a steady state and its distribution probability is given by
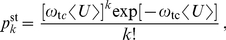
(7)which depends only on the average lifetime 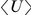 and the transcription rate 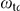.

**Figure 3:**
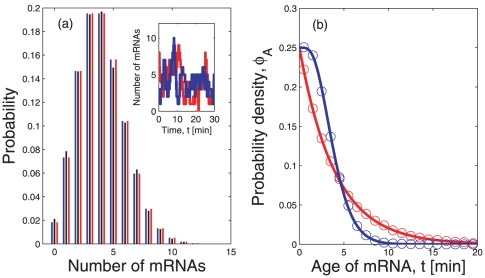
Stationary amount and age of mRNA. Panel (a) shows the histogram of the number of mRNAs for a stochastic simulation of mRNA turnover. We consider two degradation patterns, the exponential 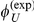 (red bars) and the gamma 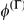 (blue bars). The black bars represent the theoretical prediction. Obviously, there are no differences between the three stationary distributions. The inset of panel (a) shows two realizations of the stochastic process of synthesis and degradation of mRNA with the two choices of the lifetime density. Panel (b) shows the age distribution of the mRNAs at steady state. Despite the fact that the stationary distributions shown in panel (a) are identical, the stationary age distributions in panel (b) are different.

In Fig. 3a, indeed, the stationary distribution 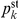 is completely independent of the form of the lifetime density and depends only on the average lifetime 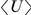. Thus, the distribution of the amount of mRNA molecules after the start of transcription is Poissonian at each time point with a parameter that depends on time. In particular, the *transient time to stationarity* depends critically on the lifetime density of the mRNA. Moreover, the transient time is large if the lifetime density is very broad. This must be particularly long in eukaryotic cells because in these organisms the average lifetime of the mRNA can be particularly long. In contrast, in prokaryotic cells, the lifetime of most mRNA molecules is relatively short so that in most of the cases the stationary state is reached within 20 minutes. The limiting (stationary) distribution given in (7) depends instead only on the mean lifetime of the mRNAs and not on any other details of the degradation process.

### The mRNA age distribution

Due to the turnover of mRNA there is an age distribution, which reflects the age composition of the mRNA pool. Also the age distribution of the mRNAs expressed after the induction evolves in time. We consider again a gene that was not transcribed before the induction. The age probability density function at time 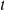 after the induction of transcription is given by
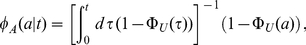
(8)where 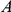 is the random variable that gives the age of a randomly chosen mRNA at time 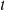 and the variable 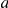 obeys 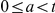 (see section *Models and Methods* for a derivation). The function 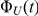 has been defined in Eq. (5). In the limit 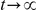 also the age distribution becomes stationary and its expression is given by
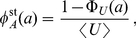
(9)which is the stationary distribution of the age of a renewal process [18]. In Fig. 3b we compare the stationary age distribution given by a gamma lifetime density 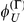 defined in Eq. (2) to the exponential case 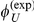 defined in Eq. (1). Clearly, in the former more young mRNAs are present, whereas in the exponential case there is a higher proportion of older mRNAs. This follows directly from the fact that the exponential distribution has a higher variance as was shown in Fig. 1a.

**Figure 4:**
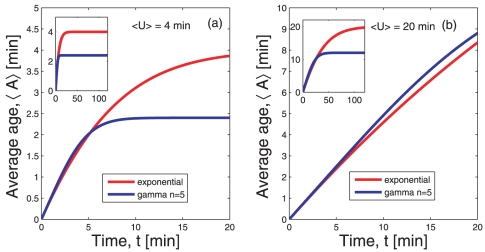
Time evolution of the average age of mRNAs. The red lines give 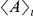 from Eq. (10) under the lifetime density 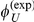 defined in (1). The blue lines give 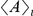 under the lifetime density 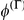 defined in (2). Panel (a) shows the time evolution of the average age of the mRNAs under the two different lifetime densities when the average lifetime is 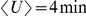. During the transient to stationarity the two average ages are very similar to each other. Conversely, at stationarity the average age under the lifetime density 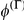 is clearly smaller than the average age under the exponential distribution 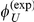. Panel (b) shows the average age as function of time for the same lifetime densities but an average lifetime 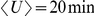. During the first twenty minutes of the transient, the average ages of the mRNAs are not very different if one compares the two distributions. However, the transient to stationarity has a different duration and at stationarity the average ages differ greatly when comparing the two lifetime densities.

The average age of the mRNAs at time 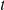 after the induction of transcription is given by
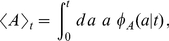
(10)and its time evolution can be followed in Fig. 4. While shortly after the induction both mRNAs have a similar average age, the effect of the different lifetime densities becomes more pronounced at larger times. The average age at steady state is lower for gamma-like mRNAs which follows from the different shape of the age distribution as shown in Fig. 3.

### The rate of protein synthesis

**Figure 5:**
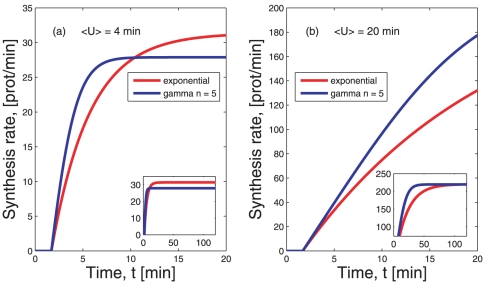
Average rate of protein synthesis as function of time. Panels (a) and (b) show the time evolution of the average protein synthesis rate 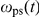 arising from the translation of the average number of mRNAs 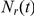 at each time point 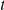, as derived in Eq. (11). The red lines represent 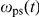 when the lifetime density is 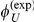 defined in (1). The blue lines depict 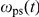 when the lifetime density is 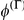 defined in (2). In both cases we have assumed a ribosome density equal to 20% of the maximal packing [23], corresponding to 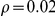 ribosomes per codon and a velocity of 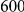 codons per minute. Panel (a) shows the change over time of the average rate of protein synthesis under the two different lifetime densities when the average lifetime is 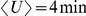. Over an initial interval of time, the rate of protein synthesis is smaller if the underlying lifetime density is exponential. At steady state the exponential lifetime leads to a larger protein synthesis rate. In (b), instead, for 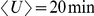 the two rates attain similar values only after about two hours. The rates are more similar than in (a) because in (b) the contribution of 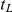 is smaller with respect to the average life time. In both plots we have fixed the length of the coding sequence 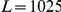 codons, corresponding to the length of the lacZ gene in *E. coli*.

Both the age distribution and the distribution of the amount of mRNA change in time depending on the shape of 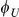. This finding has implications on the rate of protein synthesis. The protein synthesis rate in a cell is determined by the amount of mRNA and the constant ribosome flux on each mRNA. At low ribosomal densities, such as those found in *in vivo* measurements [6], [19], the average ribosome flux is given by the density 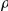 of ribosomes on an mRNA and their average elongation speed 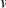. However, in the process of translation there is a transient time 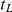 between initiation of translation and the time until the leading ribosome completes the synthesis of a protein. In eukaryotic cells and for certain prokaryotic organisms [20], [21] translation can initiate only after the whole mRNA has been synthesized. Instead, whenever translation occurs co-transcriptionally [22] translation can initiate during the synthesis of the mRNA. In both cases the transient time 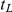 is proportional to the length of the mRNA and inversely proportional to the average elongation speed 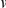, such that 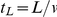. The consequence of having this transient time 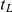 is that at any time 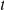 only those mRNAs that are older than 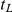 can contribute to the protein synthesis rate. Under this perspective, 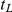 acts like a delay time that affects the rate of protein synthesis 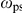, such that
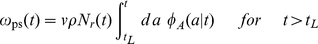
(11)and zero otherwise, with 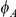 defined in (8). The rate of protein synthesis, thus, depends no both the average lifetime of the mRNA and on the form of the lifetime density (see Fig. 5).

Indeed, after the induction of transcription the protein synthesis is delayed by 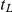 and it is followed by a transient that is governed by the increasing mRNA level, on the one hand, and by the evolution of the age distribution, on the other hand. However, ultimately both factors are due to the specific form of the lifetime density 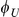. If we define 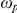 to be the degradation rate of proteins in a cell population under balanced conditions, the long time limit of (11) gives the steady state amount of proteins. This amount is given by 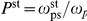, as derived for instance in [7], and this limit leads to
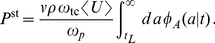
(12)Note that in principle the steady state protein level is different for the two lifetime densities since they give rise to different stationary age distributions as was pointed out in the previous section. However, this difference is small if the average lifetime is large compared to 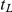. The varying rate of protein synthesis (11) has a cell biological implication. Indeed, for mRNA species that are constantly transcribed during the lifetime of the cell the amount of mRNA at the beginning of the life of the cell, namely just after cell division, will not be at the steady state level. It will reach the steady state level only if the transient to stationarity is shorter than the division time of the cell. It is thus possible that the amount of some mRNA species is never at steady state if their average lifetime 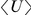 is long or if their lifetime density is very stretched. Hence, if this is the case, also the rate of protein synthesis is not constant, as shown in Fig. 5. Consequently, in a cell, the level of expression of very stable mRNAs and proteins is likely to be always away from steady state. This means that assuming steady state in a single cell may not be always accurate.

### Time evolution of polysomes and ribosomal profiles

Recently, important tools to analyze the process of translation have been developed. On the one hand, the number of ribosomes attached to each mRNA in a sample can be determined by centrifugation through sucrose gradient [19]. As a result one obtains the polysome profile that gives the distribution of the number of ribosomes translating each species of mRNA. On the other hand, even more details can be concluded from ribosomal profiling [6], [24] where also the location of the ribosomes on a species of mRNA can be determined.

Both polysome and ribosome profiles change in time since the number of ribosomes bound to an mRNA depends on the age of that mRNA. In a sample of cells, the polysome and footprinting statistics will thus depend on the age distribution of the mRNAs at the time of measurement. When the mRNA level is in steady state, this observation has been already considered in [2], [3]. However, after the induction of transcription we have to take the non-stationary age distribution into account. The analytical, or mathematical, treatment of polysomes and ribosomal profiles is possible only under several simplifying conditions. The difficulties arise due to the extended nature of the ribosomes and to exclusion events at large ribosomal densities. One can however determine the time evolution of polysomes and ribosomal profiles by running extensive computer simulations with a simple model of ribosomal walk with self exclusion (see section *Models and Methods* for details of the simulation technique).

**Figure 6:**
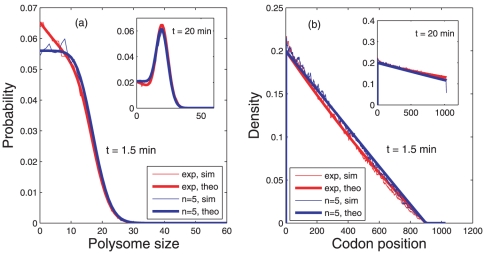
Time dependent polysome and ribosomal profiles. Panel (a) shows the distribution of the number of ribosomes on an mRNA chain of 1025 codons at two different points in time, namely after 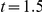 minutes and after 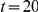 minutes. The two curves correspond to simulations based on the lifetime probability densities 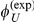 (red) and 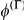 (blue), respectively. Apart for the region around small polysome sizes, both distributions lead to similar polysome profiles. However, at different time points after induction there is a noticeable change of the profiles. Thus, this demonstrates that the outcome of such an experiment depends critically on the time of measurement after the induction of that particular gene. Panel (b) shows the profile density of the ribosomes along the mRNA. The y-axis in panel (b) gives the probability that the corresponding codon in the x-axis is found covered by a ribosomes at time 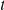. Similar to the polysome profiles, the ribosome profile densities depend on the measurement time 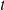 because the age composition of the sample changes with time during the transient. However, the ribosome profiles depend only weakly on the form of the underlying lifetime density 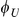 in the present case. For the simulations, we have used typical parameters determined in experiments on *E. coli*. The rate of translation initiation has been fixed to 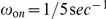 [25] and the average velocity of ribosomes is 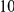 codons per second [26]. Each curve is an average over 15000 independent realizations. For all plots shown here we have taken the average lifetime 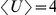 minutes. Both plots show also the predictions of the simple theory developed in [2] (solid lines) that are in remarkably good agreement with the computer simulations.

In the simulations, we have considered two different lifetime densities 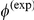 and 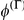 defined in (1) and (2), respectively, with an average lifetime 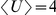 minutes and an mRNA of 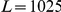 codons, corresponding to the length of the lacZ gene in *E. coli*, see Fig. 6. We find that the polysome statistics and the profile densities depend only weakly on the underlying lifetime distribution (see Fig. 6). However, both quantities depend strongly on the time of measurement following the induction of transcription. This is due to the fact that both quantities depend on the age distribution of the mRNAs in the sample, which changes with time as we have seen. Therefore, it is important to take into consideration the time after the start of induction when performing an experiment of this kind.

An additional effect of the heterogeneous age composition of the samples is given by the relatively large plateau in the polysome statistics at small polysomes [2]. This plateau depends on the form of the lifetime densities. This implies that the polysome statistics and in particular the relative amount of mRNA with small polysomes carries a signature of the degradation process of the mRNA.

## Methods

For our theoretical description, we will assume that transcription occurs at a fixed constant *population* transcription rate 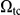. If each cell 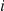, at time 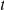, produces mRNA molecules at a rate 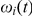, then the total transcription rate is given by 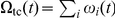. Even if the rates 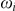 fluctuate in time in a non-synchronized way, the rate 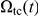 can be expressed as the sample average 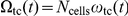 where 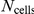 is the total number of cells under balanced conditions. If 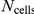 is very large, fluctuations of 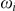 will average out and 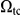 will be constant. Therefore, the process of transcription is a Poisson process with constant rate 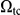. In the following, we will use the average rate 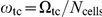 to describe the process of origination or generation of new mRNAs in an average cell.

We define 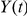 as the stochastic variable that gives the number of mRNAs at time 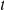. We assume that transcription starts at time zero and that the initial condition of our process is hence given by 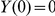. The stochastic variable 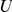 denotes the random lifetime of an mRNA molecule. The probability function 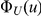 of 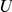 is given by
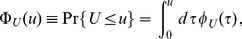
(13)where 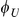 is the probability density function of 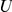. In this section we will not make any restriction concerning the form of the density 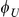 except that it must be normalized and it must have a well defined average value. Therefore, in order to leave the modeling open to any possible functional form of 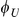, we will henceforth consider the generic form given in (13). The biochemical and theoretical considerations that allow to determine the various particular forms of the density 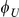 will be studied elsewhere.

In summary, we assume that mRNAs are generated at a fixed rate and live for a random time according to the probability density 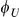. We should therefore expect that, after a certain amount of time, these two processes will balance and that the number of mRNAs 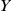 attains a stationary distribution.

### The distribution of the amount of mRNA

Let 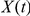 be the underlying Poisson process that describes the amount of mRNA molecules delivered to the cytoplasm until time 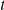 with transcription rate 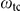,
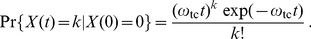
(14)We now ask for the random number of mRNA molecules 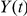 that are still present in the cell at time 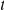. Under the general assumptions made before, we wish to compute the distribution of 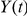, which we can formally write as
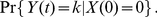
(15)Note that 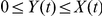 for all 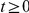. In order to determine this probability, we follow the method described in [27] chapter V section 4. The law of total probability allows us to express (15) as
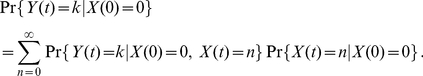
(16)We shall first recall that conditioned on the number of events up to time 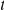, namely on 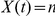, the events of a Poisson process are uniformly distributed in 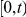 [27]. Let now 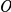 be the random origination time of a randomly chosen mRNA and let 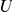 be its random lifetime. This mRNA molecule will be present at time 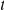 only if the variable 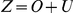 satisfies 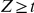. The probability 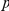 of this event gives the probability per mRNA to be present at time 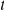 and is given by
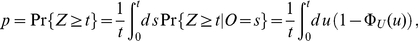
(17)where 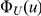 is defined in (13) and we have made use of the fact that 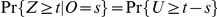. Thus, conditioned on 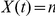, the number of mRNAs still present at time 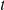 is binomially distributed according to
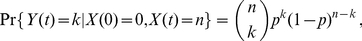
(18)with 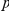 given in (17). At this point, using the law of total probability given in (16) together with (14), the time dependent distribution of the number of mRNAs at time 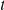 after the start of transcription is given by
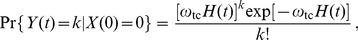
(19)where
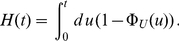
(20)Note that (19) is a time dependent Poisson distribution with parameter 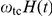. Nevertheless, one can show that 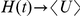 as 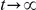 with
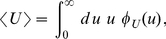
(21)being the average lifetime of the mRNA molecules. This leads to the stationary distribution
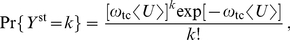
(22)which depends only on the average lifetime and not anymore on the details of the degradation process. However, as explained earlier, the time scales related to the dynamics do still depend on the form of the density 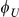.

### Age and residual life distributions

Given that there is a turnover of the mRNA, there is an age distribution of the molecules. We are interested in the age of a randomly chosen mRNA at any time point 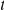. Therefore, in the following, we consider a single mRNA that has been created according to a Poisson process in the interval 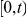 and has a random lifetime 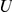 distributed according to 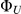. Using the same notation as before, the given mRNA will be present at time 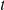 only if the variable 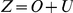 satisfies 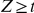. Let 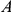 be the random variable that gives the age of a randomly chosen mRNA. Then, the age distribution of the mRNA is given by the distribution of 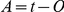 under the condition 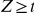. In order to compute this quantity we shall first realize that
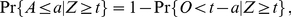
(23)and thus compute the probability density for 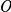 conditional that 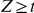. To compute this quantity, recall that, in this case, we condition that the number of transcribed mRNAs until time 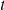 is just one. Therefore, the random variable 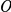 is uniformly distributed in 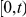. Since the transcription events are independent from another, we can thus compute the age distribution of a sample of mRNAs. Thus, we have
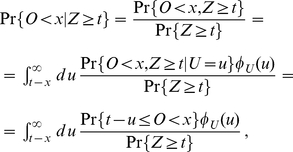
(24)where 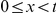 because we implicitly conditioned that the origination time point is before time 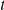. We shall now use the fact that the random variable 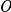 is uniformly distributed in 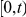 (because we have conditioned that there is one mRNA alive at time 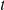) and thus that 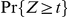 is given by (17). This leads to
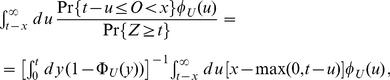
(25)from which we can compute the distribution of the age 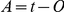 under the condition 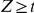. This distribution is given by (23) and reads
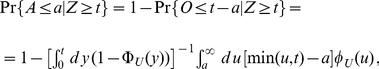
(26)which upon differentiation with respect to 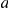 finally leads to the probability density function
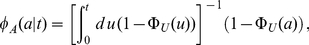
(27)for 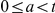 and zero otherwise.

The residual (or excess) lifetime 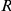 of an mRNA is a statistical quantity complementary to the age of the mRNA. The derivation of its distribution proceeds in a similar fashion as for the age distribution except from the fact that by keeping the time of origination 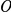 between 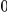 and 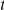, it results
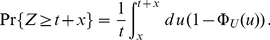
(28)Let 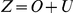 be the sum of the time origin and of the lifetime of a given mRNA and let 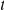 be the time of observation or measurement after the induction of transcription. The residual lifetime is given by 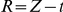. Hence, the probability distribution of the residual lifetime under the condition that 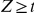 is given by
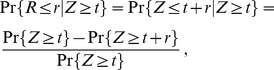
(29)which, using (17) and (28) and upon derivation by 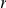, results in the probability density for 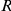


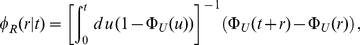
(30)for 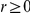. Hence, both, the age distribution as well as the residual lifetime distribution, depend on the form of the lifetime probability density 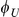 and on the time after start of transcription 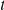.

### Computer simulations

In Fig. 6 we have simulated the motion of ribosomes along an mRNA as a simple stepping of self excluding extended objects on a linear, homogeneous chain. New ribosomes enter the initially empty mRNA only if enough space is provided. That means that the A-site of the ribosome closest to the start codon must be at least one ribosomal footprint length (10 codons) away from it. The time between two of these initiation events is exponentially distributed with an average given by the inverse initiation rate 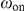 (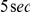). The ribosomes dwell on a codon before they move to the next one provided that it is not occupied by the preceding ribosome. The dwell times are also random with an exponential distribution and an average of 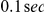. A blocked ribosome can move forward only after the preceding ribosome has left the position and a random dwell time has passed. The simulation is stopped when the mRNA has reached a predefined age. The positions of all ribosomal A-sites are recorded and further analyzed to obtain the ribosomal profile density and the polysome distribution.

We have compared these simulations to a theoretical prediction from the model developed in [2] where it was found that the time-dependent polysome statistics can be computed analytically when neglecting effects related to the mutual self-exclusion of the ribosomes. This is justified when translation initiation occurs at a small rate thus leading to a small ribosomal density. Under the same conditions, also the ribosome profile can be computed in an analytical way.

## Supporting Information

Figure S1
**Lifetime densities and evolution of the average number of mRNAs.** In panel (a) we show the lifetime density as given in Eq. (2) with different shape parameters 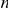 and fixed average lifetime density 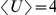 min (red: 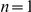, solid blue: 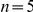, dashed blue: 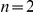, solid green: 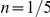, dashed green: 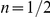). For fixed 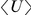 the variance of the distribution scales as 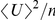. Each lifetime distribution leads to a different pattern of the growth of the mRNA number, 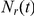, according to Eq. (6) (Panel (b)). The time to a steady state amount depends critically on the shape parameter 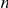 - the larger 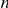 the faster a steady state is attained. Conversely, for small parameters such as 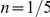 a steady state is reached only after about 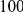 min despite an average lifetime of 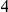 min. Note that the gamma density describes a large variety of possible mRNA lifetime distributions, although for non-integer values of the shape parameter 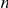 there is no clear relationship to the number of biochemical steps related to degradation.(EPS)
